# Development of Acoustic Absorbent Materials Using Pine Needles

**DOI:** 10.3390/ma18214978

**Published:** 2025-10-31

**Authors:** Jaime D. Ruiz-Martinez, Begona Peceño, Carlos J. Carrasco, Daniel Orejón, Yolanda Luna-Galiano, Carlos Leiva

**Affiliations:** 1 Departamento de Ingeniería Química y Ambiental, Escuela Técnica Superior de Ingeniería, Universidad de Sevilla, Camino de los Descubrimientos s/n, 41092 Seville, Spain; jruiz3@us.es (J.D.R.-M.); ccarrasco1@us.es (C.J.C.); yluna@us.es (Y.L.-G.); 2 Facultad de Ciencias del Mar, Escuela de Prevención de Riesgos y Medioambiente, Universidad Católica del Norte, Larrondo 1281, Coquimbo 1780000, Chile; begopc@ucn.cl; 3 School of Engineering, Institute for Multiscale Thermofluids, University of Edinburgh, The King’s Buildings, Edinburgh EH9 3FD, UK; d.orejon@ed.ac.uk; 4 Institute for Carbon-Neutral Energy Research (WPI-ICNER), Kyushu University, 744 Motooka, Nishi-ku, Fukuoka 819-0395, Japan

**Keywords:** pine needle waste, sound absorption, noise reduction composite, porosity, clogging

## Abstract

Acoustic absorbing materials made from waste plants or trees represent a sustainable source for noise reduction products and applications such as home acoustic insulation and/or traffic road noise reduction barriers. The primary aim of this work is hence to demonstrate the potential application of pine needle waste as the main constituent in acoustic absorbing materials while resin is used as binder. Once the samples have been manufactured, their different physical (density and porous structure), mechanical (compressive strength), and sound-insulating (sound absorption coefficient) properties are characterized. The influence of the ratio of pine needle/resin, length of the pine needle fragments, and thickness of the samples on the different properties has been explored. As the ratio of pine needles/resin increases so does the porosity, although the compressive strength decreases. To highlight this, the noise reduction coefficient is in the range of 0.67 and 0.71 (for 4 cm of thickness), which is higher than that reported for other typical sound absorption materials. An excess of resin produces a clogging phenomenon at the bottom of the samples, producing a reflective layer instead of an absorbent one, which could be used positively to increase the acoustic absorption coefficient in materials with combinations of sections with different needle/resin ratios. Owed to its low weight and high sound absorption coefficients at low frequencies (characteristic of road noise), PN finds usefulness in the manufacturing of environmentally friendly sound-absorbing materials as road insulation barriers.

## 1. Introduction

Each year, approximately 600 million pine trees are harvested within the European Union alone [1]. With 45% percent of these trees growing in man-made industrial forests. Pine trees are the main source of wood due to their rapid growth, which reduces costs, and are therefore widely used in construction, the furniture industry, and paper production. However, a tree represents more than its mere timber, and efficient utilization of the entire tree could help lower the demand for both wood and other natural resources.

While the trunks of pine trees are used for timber and the branches for biomass production so as to generate energy, pine needles (PN), accounting for actually 20–30% of the total tree mass, represent an unused waste as well as a potential fire risk. PN cannot be burned because of their high resin content—so billions of pine needles are left behind each year, drying or decomposing after pine trees are cut down, and are mostly responsible for forest fires when dry [1,2]. Moreover, although PN could be used as biomass to produce energy, there are several major barriers: (1) technological, (2) economic, (3) legal, (4) regulatory, (5) human resources, and (6) market-related barriers. These restrict the utility of energy generation from PN on a large scale [3].

On one hand, previous studies on pine needles have suggested their use in the production of biochar in the absorption-based removal of heavy metals from aqueous solutions [4,5], for the fabrication of nanocellulose [6,7], as reinforcing agents in polymer composites [8], and as reinforcing agent in cement [9]. Moreover, in this latter case, PN of different lengths (13, 25, and 50 mm) in amounts of 1–5 wt% can increase the cement compressive and flexural strength. Moreover, pine needle extract (PNE) is well recognized for its antioxidant properties [10] and has therefore been effectively incorporated, in varying amounts (5–20% *w*/*v*), to enhance the antioxidant activity of chitosan (CH) films [11]. While PN have also been incorporated into bio-oils/bio-chemicals, for example for bioethanol production [12,13].

On the other hand, noise pollution is another environmental issue that is growing in importance due to its detrimental effects on human health. Transportation systems comprising motor vehicles, airplanes, and trains all produce noise and are considered the primary cause of outdoor noise worldwide. Noise control via sound absorption offers a significant opportunity to investigate acoustic attenuation techniques using naturally occurring resistive materials, including cellular, fibrous, or granular structures [14,15]. To this end, materials [16,17,18,19] with higher porosity are more effective at sound absorption, as the sound waves penetrate their matrices which dissipate its acoustic energy as the sound undergoes multiple reflections within the matrices. Open pores form continuous pathways between the interior and exterior of the material, resulting in greater permeability and enhanced sound-absorbing performance, whereas, in contrast, closed pores restrict sound absorption. Key factors such as pore size, permeability, tortuosity, and thickness are essential for understanding sound absorption within material matrices [14,15,16,17,18].

Numerous sound-absorbing sustainable materials are composed of waste materials [20]. To this end, some granular waste materials, such as coal fly ashes and/or bottom ashes [21,22], metallurgical slags [23,24], construction and demolition waste [25], ceramic waste [26], recycled rubber granules [27], coffee husk and/or grounds waste [28], or mollusk shells waste [29], have been implemented as coarse aggregates in porous concrete. In these studies, the noise reduction coefficient reported (measured in an impedance tube) ranges between 0.4 and 0.6 for thicknesses between 4 and 12 cm. The low sound absorption coefficient reported can be attributed to the physical properties of those waste materials resulting in closer pore formation with the consequent rather low sound absorption reduction. Nonetheless, plant fibers or agro-industrial waste with a more elongated physical shape can be combined with resins to create high-performance sound absorption materials. These materials, such as pineapple leaf fibers [30], cotton [31], wood [32], corn fibers [33], pruning wastes [34,35], as well as other agro-industrial waste [20,36], which have thicknesses similar to those of porous concrete present higher sound absorption coefficients.

The aim of this study is hence to demonstrate the potential application of pine needles (PN) and resin as the primary components in sound-absorbing materials maximizing their sound absorption coefficient or noise reduction coefficient (NRC) while preserving satisfactory mechanical properties.

## 2. Materials and Methods

### 2.1. Materials

**Figure 1:**
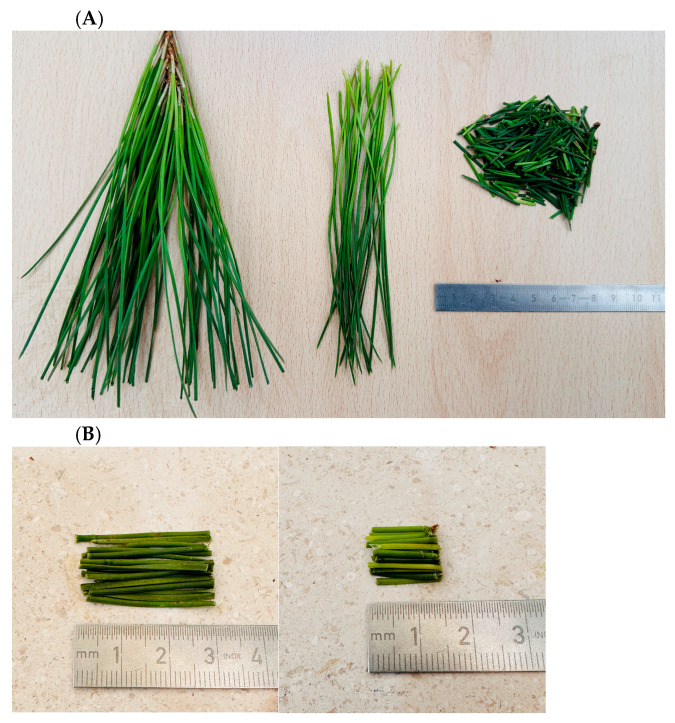
(**A**) Pine needles PN (**left**) as received, (**middle**) arranged in similar lengths, and (**right**) cut to 15 to 30 mm lengths and mixed. (**B**) Pine needles PN cut in different lengths of (**left**) 30 mm and (**right**) 15 mm.

The pine needles (PN) have a long fiber appearance, as seen in Figure 1A, but these were cut into different lengths (30 and 15 mm), as shown in Figure 1B. The PN were then used without any previous treatment except cutting.

Commercial epoxy resin (ER) (LANKOPOX 533 from PAREX S.A., Anaheim, CA, USA) compliant with EN 1504-4 [37], has been employed as the binder, which is composed of a base and a catalyst. According to previous studies for other biomass fibers [38], three different ER/PN weight ratios (1.3, 2.0, and 3.9 wt%) have been prepared and characterized for 15 mm of length and only one was manufactured with PN of 30 mm of length maintaining an ER/PN ratio of 1.30 wt%. For all compositions, samples of different thicknesses (4, 8, and 12 cm) have been manufactured.

### 2.2. Preparation of Test Specimens

After cutting the PN to the desired length, sizes of 15 mm and 30 mm, the components, including the resin base, catalyst, and the pine needles (PN), were weighed and mixed at different steps. Initially, the base and catalyst agents were mixed for 2 min. Thereafter the PN were added and kneaded for 4 min until fully impregnated within the mixture. Finally, the molds of fixed diameter (34.9 mm) were filled at different thicknesses (4, 8, and 12 cm) to produce the specimens required for the physical, mechanical, and acoustic tests. After 24 h, the specimens were removed from the molds and conditioned at ambient temperature (20 ± 2 °C) with an average relative humidity of 50 ± 2% for an additional 12 days. Following this period, the specimens were prepared for experimental testing.

**Figure 2:**
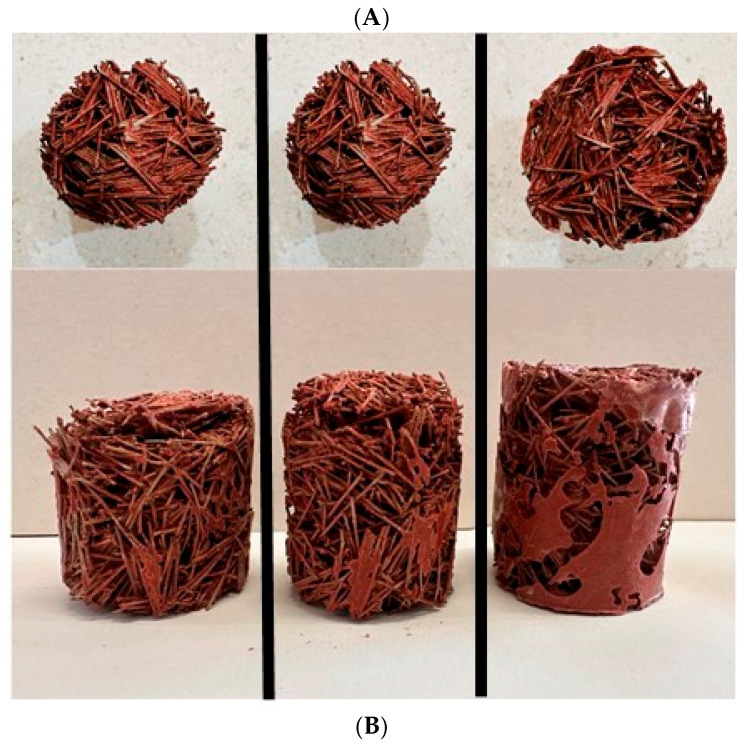
PN fibers of 15 mm samples with different ER to PN ratios of 1.3, 2.0, and 3.9 wt%. (**A**) Looking from the top or upper face and (**B**) looking from the side.

Since the hardening of the resin is not instantaneous, in the case of high resin content, it flows to the bottom of the sample, inducing a pore-clogging phenomenon [39]. Whereby the number of pores in the material’s lower part is significantly reduced, creating a gradient of porosity along the specimen thickness where the porosity in the upper part is greater than the lower part. When looking at the samples from the top, Figure 2A, the upper face presents a similar porosity interpedently of the composition or ER/PN ratio. But, when looking at the specimens from the side, Figure 2B, the porosity for the low and medium ER/PN ratio is homogeneous throughout the specimen, whereas the clogging phenomenon appears for the highest of the ER/PN ratios. The clogging phenomenon is almost non-existent for a ratio of 1.3, very limited for a ratio of 2.0, and occurring for 50% of the sample for a ratio of 3.9.

### 2.3. Test Methods

#### 2.3.1. Sound Absorption Coefficient

To determine the sound absorption coefficient and the noise reduction coefficient (NRC), the impedance tube (or Kundt tube) method was used [30]. The sound absorption coefficient at normal incidence with a total length, with the sample support, of 1.20 m was measured using a Spectronics ACUPRO impedance tube (Spectronics C., Lexington, KY, USA), a SAMSON signal amplifier (Samson Technologies, Hicksville, NY, USA), ¼ inch microphones, and the Material Testing software (ACUPRO 4 Materials Characterization Module). The measurements were carried out in a frequency range between 100 and 5000 Hz. At frequencies below 100 Hz, the flat field is not produced and the values obtained are not correct.

The 34.9 mm diameter sample is mounted at one end of a straight, rigid, smooth, and airtight impedance tube. Plane waves are generated in the tube by a sound source, and acoustic pressures are measured at two positions near the sample. The complex acoustic transfer function of the signals in the two microphones is determined and used to calculate the absorption coefficient at normal incidence and the normalized impedance of the material under test conditions.

To guarantee an appropriate fit between the tube and the sample, the circumferential edge of each test specimen was carefully sealed with petroleum jelly, in accordance with ISO 10534-2 [40]. Then, a preliminary inspection was carried out in the impedance tube to verify the adequacy of the fit, following the procedure adopted in earlier studies [31]. The measurements were performed under standard conditions (Relative humidity of 50 ± 2%, temperature of 20 ± 2 °C, and atmospheric pressure). Each value of the sound absorption coefficients reported corresponds to the mean obtained from testing three specimens of the same thickness. In addition, specimens for different ER/PN ratios (1.3, 2.0, and 3.9) and lengths (4, 8, and 12 cm) were fabricated and tested. The noise reduction coefficient (NRC) was subsequently calculated as the arithmetic average of the sound absorption coefficients at 250, 500, 1000, and 2000 Hz.

Besides the sound absorption coefficient, the acoustic absorption evaluation index (DLα) was also measured. The acoustic absorption evaluation index (DLα) is employed as a metric for the characterization of traffic noise reducing products or devices on roads, which weighs the acoustic absorption coefficient values, especially for low frequencies which are predominant in traffic noise. The determination of the DLα parameter is specified in the EN 1793-1 standard [41] and it is calculated in decibels using the following expression:

(1) $$DL\alpha =-10\times \log\left(\frac{\sum_{i=1}^{18}\alpha_{si}\times 10^{0.1\times L_{i}}}{\sum_{i=1}^{18}10^{0.1{\times L}_{i}}}\right)$$

where DLα is the acoustic absorption evaluation index and is calculated as the difference in A-weighted sound pressure levels in decibels; α_si_ is the acoustic absorption coefficient within the third octave band; and L_i_ is the A-weighted normalized sound pressure level of the road traffic noise within the third octave band of the spectrum defined in the EN 1793-1 standard in decibels. At frequencies lower than 100 Hz, the sound absorption was considered negligible (<0.05) according to previous studies [16,17,18]. The value obtained corresponds then to the acoustic absorption evaluation index DLα. The classification of absorption behavior is determined according to Table 1. Although the DLα acoustic absorption evaluation index is usually calculated with the values obtained in the acoustic absorption test in a reverberation chamber, to be able to weight the frequencies associated with road noise in this work, DLα has been calculated using the results obtained in the Kundt tube.

#### 2.3.2. Physical and Mechanical Properties

The relationship between porosity and acoustics is fundamental in the study of sound propagation and acoustic absorption in different materials. The presence of open macropores in a material can significantly influence how that material interacts with sound waves. On one hand, the bulk density (ρ) of the mortars/specimens was determined based on their weight (W) and volume (V) measurements as W/V. While on the other hand, to determine the open porosity, the samples were firstly weighted and then placed in a container with water covering all the test tubes at 20 °C for 24 h. After 24 h, the samples were taken out of the water and immediately placed in a small container, so that the water that has penetrated the inside of the sample is not lost. Samples in the container were weighed on a balance scale to determine the weight of each sample saturated with water. The open porosity (OP) was then determined according to:

(2) $$OP=\frac{{(W}_{sat}-W)/\rho_{w}}{V}100$$

where OP is the open porosity (%); W_sat_ is the weight of the specimen saturated with water; w is the weight of sample before the water immersion, ρ_w_ is density of water (998.29 kg/m^3^); and V the volume of the sample.

In addition to the open porosity, it is equally important to provide information on the pore morphology, as it is associated with the pores’ tortuosity and eventually with the material sound absorption capabilities. In this work a Nikon SMZ25 stereo microscope (Nikon Instruments Inc., Stroombaan 14, 1181 VX Amstelveen, The Netherlands) with the NIS Elements BR software Version 5.3 was used to image the 20 × 40 × 40 mm^3^ specimens.

Furthermore, the compressive strength (CS) of the specimens was evaluated in a compression-testing machine (Tinius Olsen brand, Horsham, PA, USA, Model TO317EDG) in accordance with ASTM C39/C39M-05e2 [33,42]. The compressive strength tests were performed on cylinder shape specimens of 34.9 mm in diameter and 4 cm in thickness. Each CS result represents the mean value of four experimental tests.

## 3. Results

### 3.1. Physical Properties

**Figure 3:**
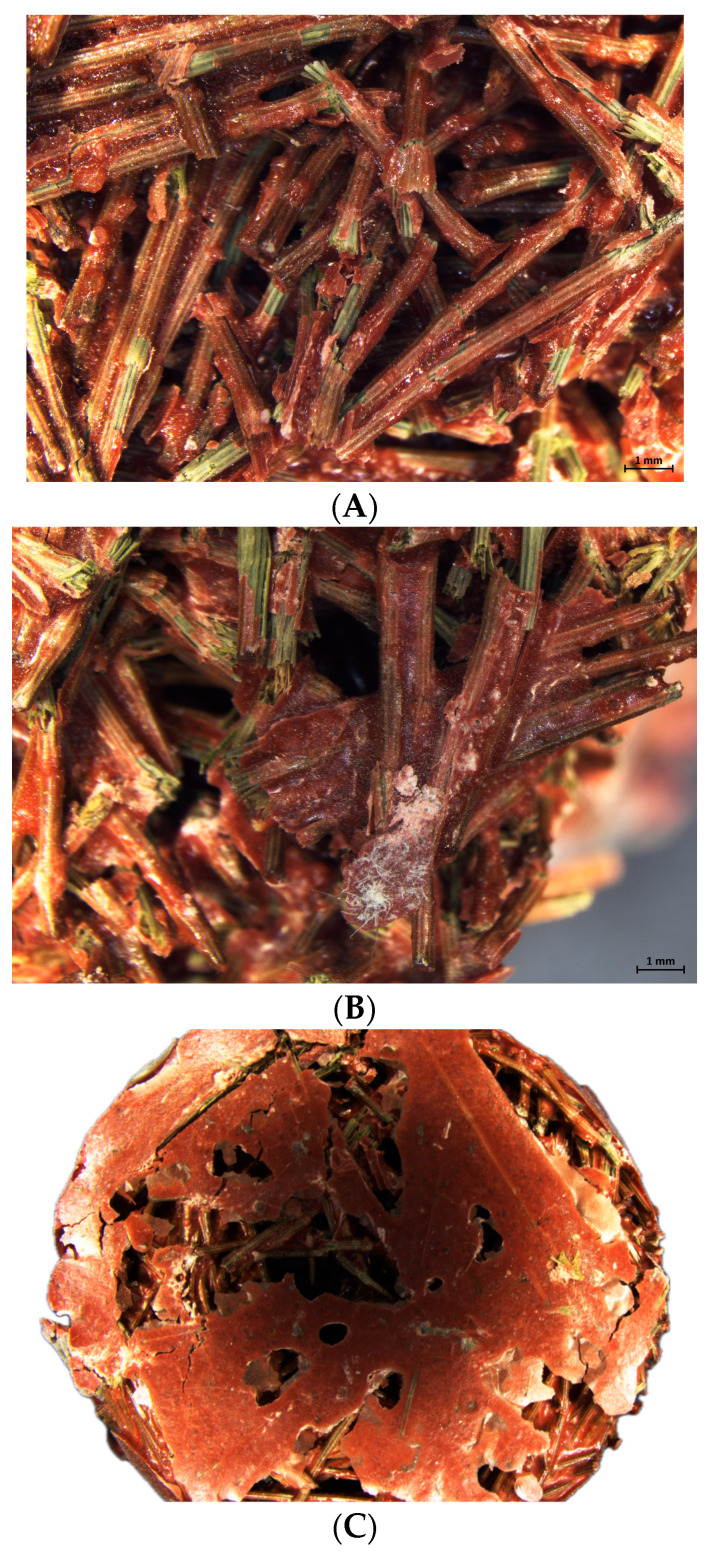
Stereo microscope images of (**A**) ER/PN = 1.3 for 15 mm of length, top section; (**B**) ER/PN = 3.9 for 15 mm of length, middle section; and (**C**) ER/PN = 3.9 for 15 mm of length, bottom section. Scale bar is 1 mm.

Figure 3 shows stereo microscope images of the samples. It can be seen how the PN are arranged in a chaotic manner with the resin giving a brownish colored finish covering the initially greenish PN. Moreover, the resin establishes junction points between the different PN, which allows the production of a solid porous matrix. As the ER/PN ratio increases, the excess of ER not only covers all the needles, but also fills the pores (Figure 3B), while at the same time the clogging phenomenon occurs at the bottom of the samples when the amount of resin is very high, i.e., for an ER/PN ratio of 3.9 (Figure 3C). Conversely, for medium and low ER/PN ratios, the pores are clearly visible and PN only agglomerates in pairs (Figure 3A) opposed to multiple PN agglomerated for the high ER/PN ratio (Figure 3B). Another consideration is that as the ER/PN ratio increases, the tortuosity of the channels increases; the resin closes only certain small channels, thereby producing less interconnection among the channels.

Table 2 presents the average and standard deviation of the open porosity (OP) and bulk density (ρ) results. It can be seen that the ER/PN ratio is inversely related to the OP of the specimens. As can be seen in Figure 3C, ER/PN-15 = 3.9 has a low open porosity and high bulk density. However, a significant difference exists between the top and bottom of the sample due to the clogging phenomena, with the top half displaying an open porosity of 42% while the bottom half yields 29%. To assess the different porosities within the same sample, two samples were cut at the height at which the clogging phenomenon occurred and then the open porosity for the two different sections were determined.

With respect to fiber length, the porosity increases with increasing length (for the same ER/PN ratio) [43,44]. Porosity results in Table 2 are in agreement with other agricultural wastes that have been utilized as sound-absorbing materials with similar resin quantities reporting porosities ranging from 50 to 80% [38,44].

On other hand, the bulk density is inversely proportional to the porosity. For the specimens made with PN 15 mm long, because of the resin’s filling effect (Figure 3) the density increases when ER/PN ratio increases. Bulk density results are also in agreement with other agro-wastes used as sound absorption materials with bulk densities ranging from 373 to 941 kg/m^3^ [43,44]. The use of long fibers produces a material of much lower density, demonstrated when comparing PN 15 mm long and PN 30 mm long, as well as with results of previous works [45].

### 3.2. Compressive Strength

**Figure 4:**
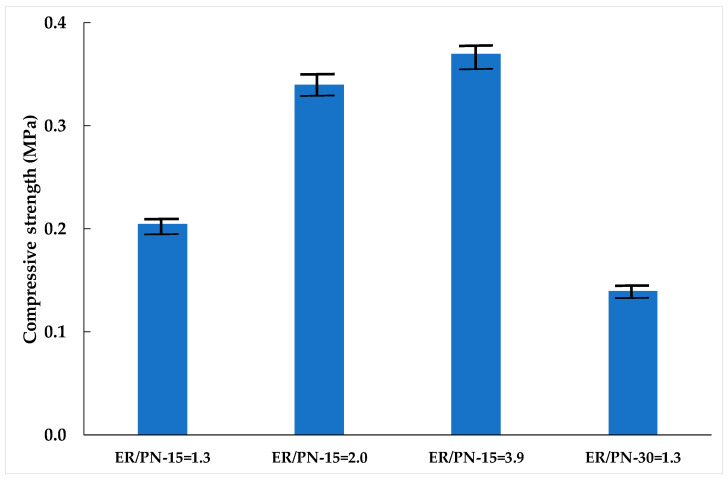
Compressive strength for different ER/PN ratios (1.3, 2.0, and 3.9 wt%) and lengths (15 mm and 30 mm).

Figure 4 presents the results of the compressive strength (CS) for the different specimens tested, which have been fabricated adopting different ER/PN ratios and PN lengths of 15 mm and 30 mm long.

The compressive strength increases as the ER/PN ratio increases. Lower ratios result in a lower compressive strength because the adhesion of the pine needle fragments by the resin is weaker in addition to higher open porosity, see Table 2. However, for higher ratios, the clogging effect produces a structurally heterogeneous material with the upper part of the sample being more porous and as such having lower compressive strength than the less-porous lower part. The compressive strength of a heterogeneous material is reported in this work as that of the weakest section. So, an increase in the ER/PN ratio from 1.3 to 3.9 does not translate into a large increase in compressive strength and only increases the compressive strength by 80% since the resin migrates towards the bottom part making the top part brittle. On the other hand, the addition of longer fibers for the same ER/PN ratio decreases the compressive strength due to the increase in open porosity and decrease in density.

The CS values are comparable to those of other materials incorporating recycled rubber granules and they are lower than composites made of rice husk and resin which have a CS of 1.2 MPa for rice/resin ratios of 1.5 [38]. Their CS is slightly lower than those observed for porous concretes using waste as coarse aggregates made of bottom ashes and mollusk shells, with construction and demolition wastes displaying a CS of 6 MPa for ratios of waste/cement equal to 4 [23,26,29].

### 3.3. Acoustic Properties

**Figure 5:**
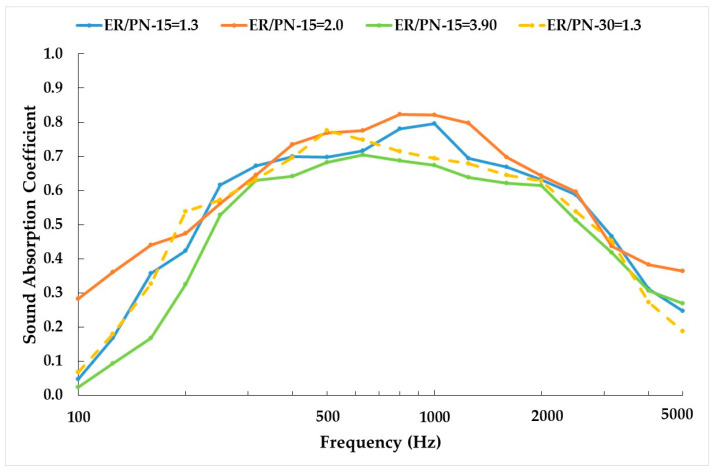
Sound absorption coefficient function of frequency (Hz) on a logarithmic scale for the different ER/PM ratios and fiber lengths.

Figure 5 illustrates the sound absorption coefficient curves obtained for the different ER/PN ratios and fiber lengths. Sound absorption coefficient curves were obtained for samples of 4 cm in thickness. Due to the clogging phenomena, the samples with the highest ER/PN ratio are placed with the most porous part on the side incident to the acoustic wave. This is to avoid sound reflection from the less porous and the closed pores present at the bottom of the sample in Figure 3C, if they were to be placed the other way around [46,47]. The sound absorption coefficient curves for the different ER/PN ratios tested generally exhibits a similar trend. The highest values are observed at low frequencies between 500 Hz and 1500 Hz in Figure 5, while at medium and higher frequencies (>1500 Hz), the sound absorption coefficient decreases.

Although the specimens prepared are made of elongated, intertwined fibers, exhibiting high porosity and high open connectivity between pores (Figure 3), the sound absorption coefficient to frequency curves reported in Figure 5 resemble the shape and behavior typical of granular materials. This is because the resin (especially for high ER/PN ratios) confer the final specimen with a rigid porous texture more alike to those found on granular materials. Hence, both the typical viscous friction dissipation and thermal loss phenomena characteristic of fibrous materials do not occur [44]. The specimens reported here exhibit therefore higher absorption at medium frequencies (500 and 1500 Hz) and lower absorption at low and high frequencies when compared to those reported in fibrous materials [17,19,44].

When sound waves propagate through the open voids of a rigid material with irregular internal geometries, the sound travels along the pores within the material, inducing vibrations in the air molecules. The sound experiences multiple internal reflections within the pore network, which extends the length and residence time of the sound wave within the material, amplifying the losses without being a direct dissipation [21].

On one hand, as the open porosity increases, so does the absorption coefficient, but an excessive increase in open porosity and pore size causes the friction between the solid frame and air to decrease, thus decreasing the acoustic absorption coefficient for low ER/PN ratio [48]. This is demonstrated when comparing ER/PN = 1.3 against ER/PN = 2.0 in Figure 5. On the other hand, as the ER/PN ratio increases, the porosity decreases and so does the sound absorption. Nonetheless, this decrease is not very significant due to the clogging phenomenon, as can be seen in Figure 5. Clogging produces a heterogeneous material, where the top 50% of the ER/PN = 3.9 specimen yields an open porosity only slightly higher than the ER/PN = 2.0 sample (see Table 1) while the bottom 50% is less porous (see Figure 2B) [49]. Despite its shorter thickness, the sound absorption provided by the top 50% of the specimen for the ER/PN = 3.9 yields a sound absorption coefficient slightly lower than the ER/PN = 2.0 sample. The less porous part does not absorb the sound but reflects it [45]. As the sound wave is reflected, it moves again through the porous section, increasing the length of the high porosity section that the sound travels through [45].

When the fiber size increases (maintaining a constant ER/PN ratio), the open porosity increases considerably and therefore the absorption values decrease due to the diminution of internal sound reflections, especially at higher frequencies. However, the peak corresponding to the maximum absorption shifts towards lower frequencies (from 1000 Hz for 15 mm length to 500 Hz for 30 mm length).

Next, Table 3 shows the results obtained from the NRC and DLα that follow a similar trend when comparing the type of specimen/composition. According to DLα only ER/PN-15 = 3.9 can be classified as A1 while the others are classified as A2.

Compared to other studies using fibrous materials of similar thickness such as cane, sisal, kenaf, and/or coconut, the sound absorption coefficient is superior at low frequencies (>0.4) but lower at medium and high frequencies (<0.8) than for these similar materials [50,51,52]. On the other hand, the sound absorption coefficients reported for the PN are higher than porous concretes using wastes as coarse aggregates [23,26,29] at all frequencies tested. Although both materials are rigid porous materials, the porous network with the PNs presents as more interconnected and with more tortuosity than in porous concretes.

**Figure 6:**
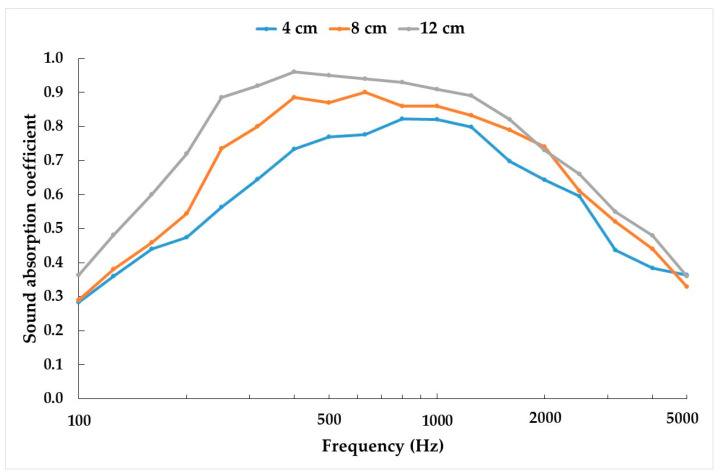
Sound absorption coefficient function of frequency (Hz) for the different thicknesses (4, 8, and 12 cm) of ER/PN-15 = 2.0.

Next, the sound absorption coefficient for the various specimen thicknesses (4, 8, and 12 cm) for ER/PN = 2.0 are presented in Figure 6. As the specimen length increases so does the maximum sound absorption coefficient peak. In addition, the frequency at which the first maximum absorption peak occurs shifts to lower frequency values, which is the characteristic behavior of porous materials on rigid walls [53]. This behavior is attributed to the increased tortuosity and length of the channels through which the sound waves propagate and reflect, leading to greater energy dissipation and to a greater sound absorption coefficient [54].

Table 4 shows the results obtained for the NRC and DLα for ER/PN-15 = 2.0 and different thicknesses. It can be seen that as the thickness increased so did both the NRC and specially DLα, because increasing the thickness increases the sound absorption at low frequencies.

**Figure 7:**
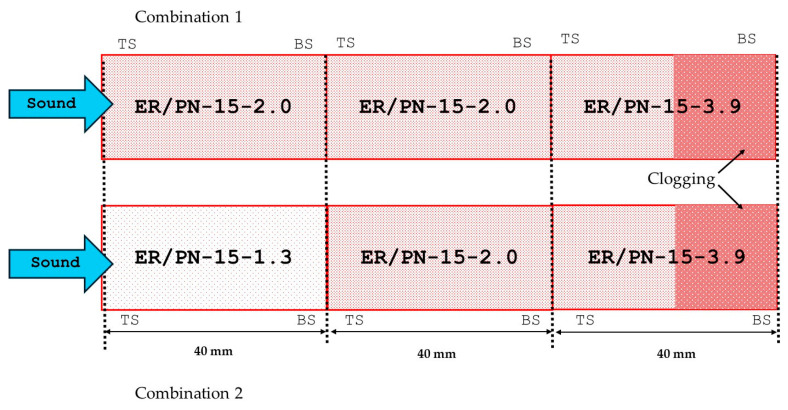
Combination of the three different ER/PN materials placed in series. (Combination 1 and Combination 2). Note: TS = top side during the fabrication process, BS = bottom side during the fabrication process. Differences in the shaded red regions provide a qualitative indication of the sample porosity.

Finally, different combinations of the three different ratios have been analyzed by placing three different test pieces of 40 mm of the same PN lengths but of different ER/PN ratios for a total length of 120 mm, as shown in Figure 7.

**Figure 8:**
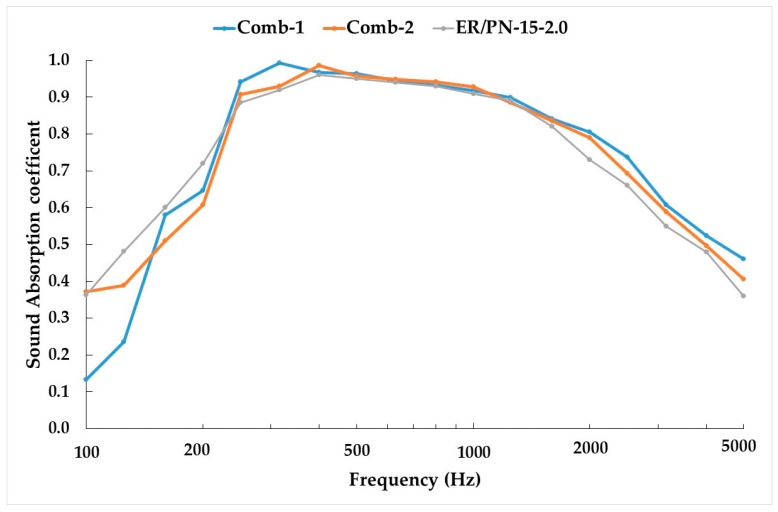
Sound absorption coefficient function of the frequency (Hz) for the different combinations (Combination 1, Combination 2) and comparison to uniform ER/PN-15-2.0 with 12 cm of thickness.

Figure 8 shows the sound absorption coefficient function of the frequency for two different combinations of three specimens placed in series. On one hand, as shown in Figure 8, due to the clogging effect, the addition of a less-porous section at the end of the series where 5/6ths of the section display a similar porosity (Combination 1) results in a slight increase in the maximum sound absorption peak between 200 and 400 Hz. Also, at frequencies above 1500 Hz the sound absorption coefficient curve is above the other two samples tested (Combination 2 and uniform ER/PN-15-2.0) for comparison. This effect produces an increase of 5% in the NRC and 4.3% with respect to the DLα (Table 5) with respect to when the entire material is composed of the one with the highest absorption coefficient (ER/PN-15-2.0 in Table 3). Combination 1 can be classified as A3 according to Table 1. As the sound wave reaches the last part of the section in series, the shock wave is forced to bounce back as it encounters the clogging section with less pores ER/PN-15-3.90. The sound wave hence travels a greater distance with the consequent enhanced sound absorption [22,49,55].

On the other hand, when the sample is made up of a first section with a low ER/PN ratio, a second section with a medium ER/PN ratio, and a third section with the highest ER/PN ratio (Combination 2), the frequency for the maximum acoustic absorption coefficient values shifts slightly to lower frequency values between 250 and 450 Hz. The sound absorption coefficient values at those frequencies increase when compared to the uniform ER/PN-15-2.0. Furthermore, higher sound absorption coefficients are achieved at frequencies above 1500 Hz for both Combination 1 and Combination 2 when compared to uniform ER/PN-15-2.0 (Figure 8). As indicated by the data presented in Table 5, Combination 2 presents a 6% and 7% increase in NRC and DLα, respectively, compared to the uniform ER/PN-15-2.0 for 12 cm of thickness. Combination 2 can be classified as A3 according to Table 1. Owed to the three different porosities displayed within Combination 2, the first section absorbs at different frequencies than the second, and a further change in porosity between the second section and the third section, and at the middle of the third section, increases tortuosity and noise reflection at the end of the specimen, eventually favoring the dissipation of sound energy across different frequencies.

## 4. Conclusions

The fabrication and characterization of sustainable sound-absorbing materials from waste pine needles (PN) making use of epoxy resin as the binder have been studied. The findings are as follows:

- Concerning the effect of the resin content, the porosity is found to be inversely proportional to the amount of resin, and as such the density is then directly proportional to the resin content. However, for large quantities of resin a clogging phenomenon occurs that produces a material with heterogeneous physical properties. Regarding the mechanical properties, it was determined that specimens with a greater amount of resin have a higher compression strength, but the clogging effect limits the increase in compressive strength for high ratios. An 80% higher compressive strength is reported for ER/PN-15-3.9 compared to ER/PN-15-1.3). While the sound absorption coefficient for the different ratios increases when the ratio ER/PN decreases. A 10% higher sound absorption coefficient is reported for ER/PN-15-1.3 when compared to ER/PN-15- = 3.9. The clogging effect on the other hand produces an absorbent section and a reflective section, which makes the decrease in the acoustic absorption coefficient not as high as one would expect.
- Regarding the influence of the length of pine needle fragments, the porosity of the specimens is directly proportional to the length of the pine needle. Hence increasing the pine needles length increases the porosity in turn decreasing the density, which eventually has a negative effect on the compressive strength. However, the sound absorption coefficient does not change significantly. A 2% difference is found when comparing ER/PN-15-1.3 and ER/PN-30-1.3.
- With respect to the influence of thickness, the greater thickness of the specimens leads to improved sound absorption coefficients. For the same composition, i.e., uniform, ER/PN-15-2.0 provides an 18% greater sound absorption coefficient when extending the length from 4 to 12 cm. This is because sound waves travel a longer path and hence are more effectively absorbed by the material. The tests for the ER/PN-15-2.0 yield the best sound absorption coefficient for the longest of the specimens at 12 mm.
- Regarding the influence of combining different ratios, an increase in the sound absorption coefficient at medium and high frequencies was observed, which is attributed to the presence of clogging at the end of the high ER/PN ratio specimens. While at lower frequencies the specimen compressing different ER/PN ratios of 1.3/2.0/3.9 placed in series presents the best acoustic properties. A 6% and a 7% increase for NRC and DLα, respectively, are reported when compared to the uniform ER/PN-15-2.0 for 12 cm of thickness. Both combinations of ER/PN ratios can be classified as A3.

Given their low weight and high absorption coefficient values reported at low frequencies (characteristic of typical road noise), this material finds excellent potential use as sound absorbent material inside metallic road noise barriers, which do not require high mechanical properties.

## Figures and Tables

**Table 1:** Absorption classification according to the acoustic absorption evaluation index DLα [41].

Category	DLα (dB)
A0	Undetermined
A1	<4
A2	4 to 7
A3	8 to 11
A4	>11

**Table 2:** Physical properties of open porosity and bulk density for different ER/PN ratios and PN lengths.

Composition	Open Porosity (%)	Bulk Density (kg/m^3^)
ER/PN-15 = 1.3	64 ± 4	645 ± 14
ER/PN-15 = 2.0	46 ± 3	786 ± 22
ER/PN-15 = 3.9	31 ± 3	883 ± 32
ER/PN-30 = 1.3	75 ± 4	331 ± 11

**Table 3:** Noise reduction coefficient (NRC) and acoustic absorption evaluation index DLα (dB) for 4 cm of thickness.

Composition	NRC	DLα (dB)
ER/PN-15 = 1.3	0.69 ± 0.03	4.7 ± 0.2
ER/PN-15 = 2.0	0.71 ± 0.03	5.3 ± 0.2
ER/PN-15 = 3.9	0.63 ± 0.03	3.9 ± 0.2
ER/PN-30 = 1.3	0.67 ± 0.04	4.4 ± 0.3

**Table 4:** Sound absorption properties NRC and DLα for different thicknesses for the ER/PN-15 = 2.0 specimen.

Thickness (cm)	NRC	DLα (dB)
4	0.70 ± 0.03	5.3 ± 0.2
8	0.76 ± 0.03	6.2 ± 0.2
12	0.85 ± 0.04	7.8 ± 0.4

**Table 5:** Sound absorption properties NRC and DLα for the different combinations tested.

Sample	NRC	DLα (dB)
2.0-2.0-3.9	0.90 ± 0.03	8.0 ± 0.2
1.3-2.0-3.9	0.91 ± 0.03	8.3 ± 0.4
Uniform ER/PN-15-2.0		

## Data Availability

The original contributions presented in the study are included in the article. further inquiries can be directed to the corresponding author.
